# Schwere kutane Arzneimittelreaktionen bei Kindern

**DOI:** 10.1007/s00112-023-01753-3

**Published:** 2023-04-18

**Authors:** Maja Mockenhaupt

**Affiliations:** Dokumentationszentrum schwerer Hautreaktionen (dZh), Universitätsklinik für Dermatologie und Venerologie, Hauptstraße 7, 79104 Freiburg, Deutschland

**Keywords:** Epidermale Nekrolyse, „Drug reaction with eosinophilia and systemic symptoms“, Antikonvulsiva, Sulfonamide, Sulfasalazin, Epidermal necrolysis, Drug reaction with eosinophilia and systemic symptoms, Anticonvulsants, Sulfonamides, Sulfasalazine

## Abstract

Schwere kutane Arzneimittelreaktionen kommen auch bei Kindern vor und reichen von blasenbildenden Reaktionsformen der Haut und Schleimhaut bis zu ausgedehnten Exanthemen mit Blutbildveränderungen und Beteiligung innerer Organe. Zu den erstgenannten gehören das Stevens-Johnson-Syndrom (SJS) und die toxische epidermale Nekrolyse (TEN), die als eine Krankheitsentität mit verschiedenen Schweregraden angesehen und auch als „epidermale“ oder „epitheliale Nekrolyse“ (EN) zusammengefasst werden. Die Gruppe der Arzneimittelreaktionen mit primär systemischen Veränderungen wird durch eine als „drug reaction with eosinophilia and systemic symptoms“ (DRESS) bezeichnete Reaktionsform repräsentiert.

Obwohl die EN allgemein als Arzneimittelreaktion gilt, lässt sich bei Kindern nur in der Hälfte der Fälle ein medikamentöser Auslöser ausmachen. Erst nach einer klaren Diagnosestellung sollten spezifische therapeutische Maßnahmen folgen, wobei das Absetzen des auslösenden Agens bei arzneimittelinduzierten Fällen die entscheidende Rolle spielt. Um das verursachende Arzneimittel identifizieren und absetzen zu können, muss eine sehr detaillierte Arzneimittelanamnese erhoben werden. Zu den hochverdächtigen Auslösern von EN und DRESS bei Kindern gehören bestimmte Antiepileptika, Sulfonamide und Sulfasalazin. Die supportive Therapie mit entsprechenden Lokalmaßnahmen, Schmerztherapie, augenärztlicher Mitbetreuung etc. ist bei EN unersetzlich, doch hat sich eine kurzzeitige immunmodulierende Therapie mit Cyclosporin A als hilfreich erwiesen. Bei DRESS hingegen wird eine mittel- bis längerfristige systemische Therapie mit Glukokortikosteroiden empfohlen.

## Abstract (en)

Severe cutaneous drug reactions also occur in children and range from reactions with blister forming of skin and mucosa to extensive exanthems with altered differential blood count and involvement of internal organs. The first group includes Stevens-Johnson syndrome (SJS) and toxic epidermal necrolysis (TEN), which are considered as one disease entity with different degrees of severity and are also referred to as “epidermal” or “epithelial necrolysis” (EN). The group of drug reactions with primarily systemic alterations is represented by a condition known as drug reaction with eosinophilia and systemic symptoms (DRESS).

Although EN is generally considered as a drug reaction, a causative agent can only be identified in about 50% of all pediatric cases. Once a clear diagnosis is established, specific treatment measures should be carried out, whereby discontinuation of the causative agent plays a decisive role in drug-induced cases. In order to be able to identify and discontinue the drug responsible, a detailed medication history must be obtained. Certain antiepileptic drugs, sulfonamides and sulfasalazine are among the most frequent triggers of EN and DRESS in children. Supportive therapy including appropriate topical treatment, pain management and ophthalmological consultations are of utmost importance in EN but a short-term immunomodulating treatment with cyclosporine A has been shown to be helpful. In contrast, in DRESS middle to long-term systemic treatment with glucocorticosteroids is recommended.

Schwere kutane Arzneimittelreaktionen können in jedem Lebensalter, bei beiden Geschlechtern und bei Menschen unterschiedlicher ethnischer Herkunft auftreten. Hierzu gehören blasenbildende Haut- und Schleimhautreaktionen wie das Stevens-Johnson-Syndrom (SJS) und die toxische epidermale Nekrolyse (TEN), die aufgrund des klinischen Bildes sowie der gemeinsamen Pathogenese und Ätiologie als eine Krankheitsentität unterschiedlichen Schweregrads als epidermale oder epitheliale Nekrolyse (EN) zusammengefasst werden. Hiervon wird die „drug reaction with eosinophilia and systemic symptoms“ (DRESS) unterschieden; diese geht mit einer Beteiligung innerer Organe einher.

## Häufigkeit und Demografie

### Epidermale Nekrolyse

Mit einer Inzidenz von einem bis zwei Fällen/1 Mio. Personen und Jahr ist die EN insgesamt sehr selten. Bei Kindern kommt diese Reaktionsform deutlich seltener vor als bei Erwachsenen, wie in einer populationsbezogenen Untersuchung des Dokumentationszentrums schwerer Hautreaktionen (dZh an der Klinik für Dermatologie des Universitätsklinikums Freiburg) mithilfe von Daten des Statistischen Bundesamtes in Deutschland gezeigt werden konnte [18, 26]. Das Sterberisiko ist bei Erwachsenen sehr hoch und steigt mit dem Ausmaß der Blasenbildung, dem Alter der Patienten und den vorliegenden Grunderkrankungen [29] bis ca. 9 % bei SJS und ca. 48 % bei TEN (für alle Schweregrade zusammengenommen ca. 22–29 %, [15]). Für Kinder liegen kaum verlässliche epidemiologische Daten vor; es wird von einer Letalität von ca. 6 % der betroffenen Kinder und Jugendlichen unter 18 Jahren ausgegangen [18, 20].

Die geschätzte Letalität der von EN betroffenen Patienten unter 18 Jahren beträgt ca. 6 %

Allerdings geben publizierte Fallberichte häufig ein verzerrtes Bild, da Kasuistiken über schwere Verlaufsformen bei Kindern mit gutem Ausgang eher veröffentlicht werden als die letal ausgehenden Krankheitsfälle alter Menschen. Die oben genannte Analyse von validierten Fällen des dZh nach Altersgruppen ergab, dass die Inzidenz von EN bei Erwachsenen deutlich höher ist als bei Kindern und Jugendlichen (3/Mio. Patienten im Alter ≥ 80 Jahre vs. 0,36/Mio. Patienten im Alter < 12 Jahre) und die meisten Fälle in Regionen auftreten, in denen vorwiegend ältere Menschen leben. Gründe könnten eine erhöhte Arzneimitteleinnahme im höheren Lebensalter sein, doch könnten auch andere, bislang unklare Ursachen infrage kommen [18].

### „Drug reaction with eosinophilia and systemic symptoms“

Bislang existieren keine verlässlichen Daten zur Inzidenz von Drug reaction with eosinophilia and systemic symptoms (DRESS). Früher Angaben, die immer wieder zitiert werden, beziehen sich auf den Terminus „Hypersensitivitätssyndrom“, der unterschiedliche schwere Unverträglichkeitsreaktionen auf Arzneimittel subsumierte. Die angegebenen Häufigkeiten variierten stark (1:1000 bis 1:10.000) und bezogen sich v. a. auf Reaktionen nach Einnahme von Antiepileptika. Die Letalität wurde auf 10 % geschätzt; als Todesursache wurde meist Leberversagen genannt. Da populationsbezogene Untersuchungen mit hoher Erfassungsrate wie bei EN fehlen – auch weil DRESS oft nicht als eigene Reaktionsform erkannt wird –, sind auch heutige Inzidenzschätzungen für DRESS nicht sicher. Allerdings konnte anhand streng validierter DRESS-Fälle gezeigt werden, dass die Letalität mit 2 % deutlich niedriger als die zuvor angenommene ist [10].

Zu DRESS bei Kindern liegen zwar Fallserien, aber keine spezifischen epidemiologischen Untersuchungen vor. Die Analyse von 117 streng validierten DRESS-Fällen ergab, dass etwas mehr Frauen als Männer erkrankt waren, wobei die betroffenen Frauen signifikant jünger waren als die Männer (Median 44 Jahre vs. 56 Jahre). Eine mögliche Erklärung könnte sein, dass manche der auslösenden Medikamente v. a. bei jungen Frauen im gebärfähigen Alter eingesetzt werden, wie z. B. Lamotrigin. Aber möglicherweise spielen weitere bisher unbekannte Faktoren eine Rolle [10].

## Klinisches Bild, diagnostische Maßnahmen und Differenzialdiagnosen

### Epidermale Nekrolyse

**Figure 1:**
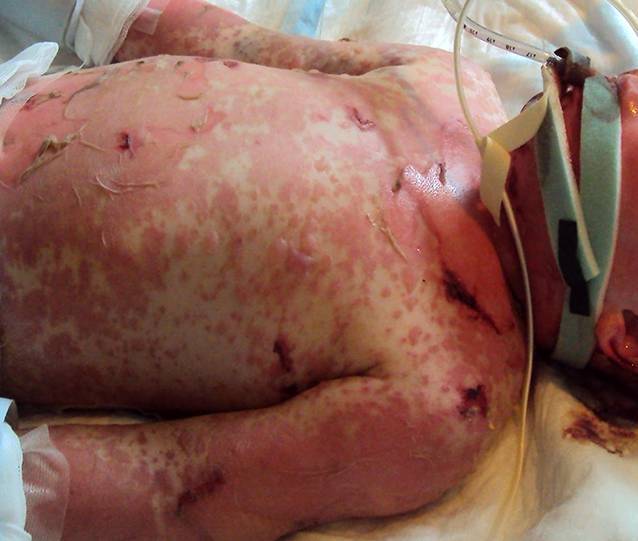

**Figure 2:**
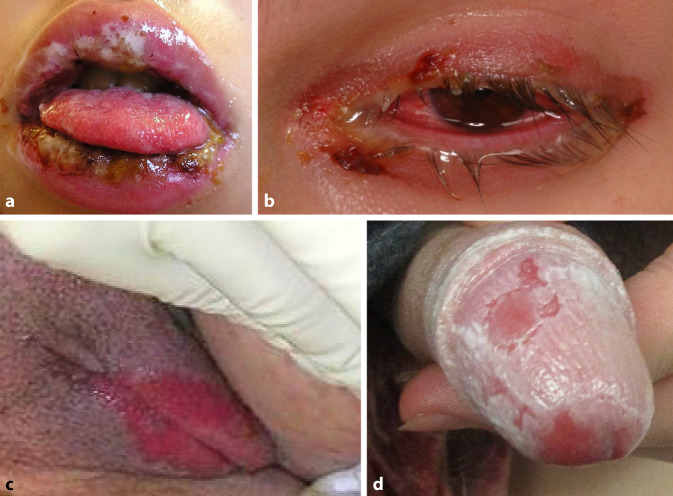

An der Haut finden sich fleckige und kokardenförmige Erytheme mit Übergang in eine z. T. ausgedehnte Blasenbildung, die einer großflächigen zweitgradigen Verbrennung oder Verbrühung ähneln kann (Abb. 1). Daher rührt auch der Begriff „Syndrom der verbrühten Haut“, der allerdings nicht mit dem „staphylococcal scalded skin syndrome“ (SSSS) verwechselt werden darf. Zudem imponieren erosive Schleimhautläsionen oral, labial, konjunktival und genital (Abb. 2a–d); auch Nasen‑, Anal- oder Bronchialschleimhaut können betroffen sein. Meist liegen gleichzeitig Fieber und oft ein ausgeprägtes Krankheitsgefühl vor [15, 16].

Aufgrund einer bereits 1993 veröffentlichten Konsensusdefinition werden die verschiedenen EN-Schweregrade (SJS < 10 % Hautablösung; SJS/TEN-Übergangsform 10–30 %; TEN > 30 %) unterschieden und vom Erythema exsudativum multiforme majus (EEMM) differenziert (Tab. 1; [3]).

**Table 1:** 

Einteilung	EEMM	SJS	SJS/TEN-Übergangsform	TEN mit Maculae (Flecke)	TEN auf großflächigen Erythemen (Ohne Flecke)
Hautablösung	< 10 %	< 10 %	10–30 %	> 30 %	> 10 %
Typische Kokarden	Ja	–	–	–	–
Atypische Kokarden	Erhaben	Flach	Flach	Flach	–
Maculae (Flecke)	–	Ja	Ja	Ja	–
Verteilung	Extremitätenbetont	Stammbetont/generalisiert	Stammbetont/generalisiert	Stammbetont/generalisiert	Stammbetont/generalisiert

*EEMM* Erythema exsudativum multiforme majus, *SJS* Stevens-Johnson-Syndrom, *TEN* toxische epidermale Nekrolyse

Als wichtiges klinisches Zeichen sollte geprüft werden, ob sich in den betroffenen Hautarealen die Oberhaut verschieben lässt, d. h., das Nikolski-Phänomen positiv ist. Unterschieden wird das direkte Nikolski-Phänomen, bei dem sich die Epidermis durch tangentialen Fingerdruck infolge der epidermalen Kohärenzschädigung „abschieben“ lässt, vom indirekten Nikolski-Phänomen, bei dem eine bereits bestehende Blase „weitergeschoben“ werden kann [15]. Zudem kann zwischen dem „feuchten“ und „trockenen“ Nikolski-Phänomen differenziert und anhand der klinischen Beschaffenheit des Blasengrundes auf die Höhe der Spaltbildung geschlossen werden [16, 21]. Verlässlicher für die Differenzierung zwischen SSSS und EN ist sicherlich die Schnellschnittdiagnostik eines Kryostatpräparates. Bei vorliegendem SSSS weist das entnommene Blasendach eine subkorneale Spaltbildung und bei EN eine tiefer liegende subepidermale Spaltbildung auf. Dennoch sollte möglichst in allen Fällen von schweren Hautreaktionen eine Probebiopsie zur konventionellen histologischen Aufarbeitung aus dem erythematösen Randbereich der blasigen Läsionen erfolgen, damit sowohl Epidermis- als auch Dermisanteile erfasst werden [32]. Dies gilt auch für Kinder, bei denen aus Furcht vor einer Narbenbildung häufig auf eine Biopsie verzichtet wird. Angesichts der Schwere der Erkrankung und der Risiken einer Fehldiagnose sind Risiko und Folgen einer 4‑mm-Stanzbiopsie zu vernachlässigen. Jeder Sturz im Kindesalter kann ausgedehntere Narben hinterlassen als eine solche diagnostische Hautprobe.

**Figure 3:**
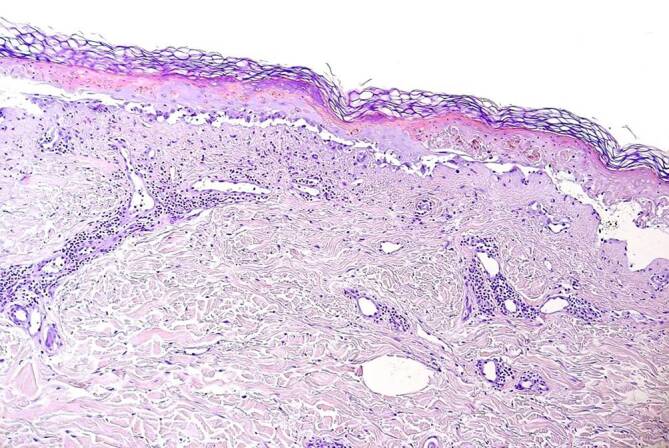

Die histologische Aufarbeitung von EN zeigt nekrotische Keratinozyten in disseminierter Verteilung bis hin zur kompletten EN. Zudem imponiert eine Vakuolisierung der Basalmembranzone bzw. eine subepidermale Spalte. In der oberen Dermis fällt ein perivaskuläres lymphohistiozytäres Infiltrat, in dem auch eosinophile Granulozyten vorkommen können, auf (Abb. 3 [32]). Derselbe histologische Befund findet sich beim EEMM, v. a. wenn die Biopsieprobe der zentralen Blase einer Kokardenläsion genommen wird. Auch beim generalisierten bullösen fixen Arzneimittelexanthem (GBFAE) findet sich der beschriebene histologische Befund, obwohl hier manchmal mehr Eosinophile zu entdecken sind und bei Wiederholungsereignissen eine dermale Pigmentablagerung auffällt. Eine klare Unterscheidung zwischen den genannten Krankheitsentitäten lässt sich nur in der Zusammenschau von klinischem Bild und histologischem Befund treffen. Während die Nekrose der Epidermis bei EN, EEMM und GBFAE zur Ablösung führt, hebt sich bei anderen bullösen Dermatosen hingegen zunächst vitale Epidermis ab; diese wird erst sekundär nekrotisch, was sich allerdings nur mithilfe früher Biopsien erkennen lässt. Falls differenzialdiagnostisch autoimmunologische bullöse Dermatosen in Betracht gezogen werden, sollte zusätzliche eine Immunfluoreszenzuntersuchung erfolgen [16, 32]. Spezifische Laborparameter, die für die Diagnose EN sprechen, fehlen.

**Figure 4:**
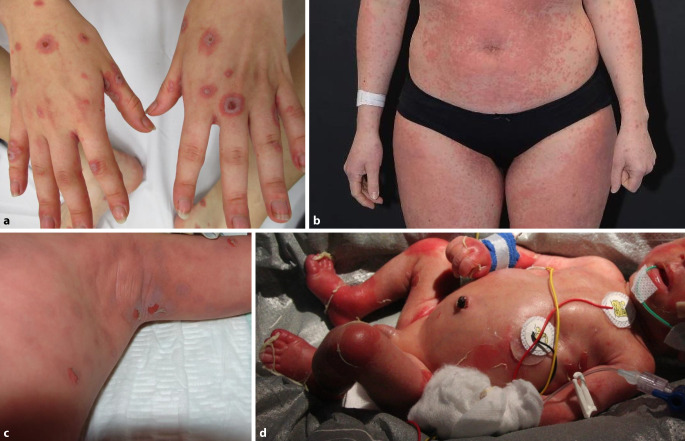

Eine wichtige Differenzialdiagnose der EN (besonders der weniger ausgedehnten Form SJS) im Kindes- und Jugendalter ist das EEMM. Es handelt sich nicht – wie fälschlicherweise oft geschrieben steht – um eine Form des Stevens-Johnson-Syndroms (SJS), sondern um eine eigene Entität [1]. Beim EEMM imponieren typische schießscheibenförmige Läsionen, sog. Kokarden (Abb. 4a), bei gleichzeitigem Vorliegen einer erosiven Schleimhautbeteiligung. Bei Kindern und Jugendlichen finden sich überwiegend typische Kokarden und/oder atypische Kokarden sowie „Riesenkokarden“ in stammbetonter oder generalisierter Verteilung („untypisches EEMM“), während die Kokarden bei Erwachsenen meist akral lokalisiert sind („typisches EEMM“, [25, 28]). Diese Läsionen können ineinander übergehen, lassen sich jedoch immer noch gut von der nichtbetroffenen Haut abgrenzen. Mögliche Übergänge in ein konfluierendes Exanthem mit großflächiger Blasenbildung wie bei EN sind nicht zu erwarten, was nicht nur therapeutisch, sondern auch hinsichtlich der Prognose des Patienten von Bedeutung ist. Aufgrund der vorliegenden erosiven Schleimhautläsionen können EEMM und EN nicht unterschieden werden, da diese bei beiden Reaktionsformen vorhanden sind.

Das EEMM und die EN unterscheiden sich nicht nur im klinischen Bild, sondern auch in ihrer Ätiologie. Während das typische EEMM (und auch die Minorform EEM ohne Schleimhautbeteiligung) häufig durch akute Herpes-simplex-Virus-Infektionen und -Reaktivierungen induziert ist, wird das untypische EEMM v. a. im Kindes- und Jugendalter durch andere Infektionen, wie z. B. Mykoplasmenpneumonie, sonstige Infektionen der oberen Luftwege, Influenza und virale Atemwegsinfektionen ausgelöst. In ca. 10–20 % der Fälle ist ein wiederholtes Auftreten möglich. Das EEMM ist keine Arzneimittelreaktion, sondern eine „hypererge“ Reaktion auf eine Infektion [1, 25].

Zu Beginn der Reaktion, v. a. wenn das Vollbild der EN noch nicht vorliegt, gehören makulöse und vesikulöse Exantheme verschiedener Genese zu den Differenzialdiagnosen, zumal gerade bei Kindern viele Virusexantheme mit einer Schleimhautbeteiligung einhergehen. Nicht selten wird bei Auftreten eines vesikulösen Hautausschlags mit oraler Schleimhautbeteiligung und Fieber der Verdacht auf Varizellen geäußert. Bei vorrangiger Schleimhautbeteiligung kommt differenzialdiagnostisch die Hand-Fuß-Mund-Krankheit, ausgelöst durch eine Infektion mit Coxsackie-Viren in Betracht. Auch anuläre, manchmal urtikarielle Effloreszenzen, die an Kokarden erinnern, können auffallen (Abb. 4b). Die entsprechenden Abstriche und serologischen Untersuchungen sollten in solchen Fällen auf den Weg gebracht werden [25].

Eine weitere bedeutende Differenzialdiagnose der EN ist das GBFAE, bei dem sich multiple bis handflächengroße, bräunlich livide Plaques zeigen, auf denen schlaffe Blasen entstehen (Abb. 4c). Die Blasenbildung beträgt meist weniger als 10 % der Körperoberfläche (KOF), und das Nikolski-Phänomen auf nichterythematöser Haut ist negativ. Oftmals lässt sich eine ähnliche, teilweise lokalisierte Reaktion in der Vorgeschichte eruieren. Die Patienten befinden sich in einem deutlich besseren Allgemeinzustand als EN-Patienten, und sofern eine Schleimhautbeteiligung vorliegt, ist diese leicht ausgeprägt und betrifft nicht die Konjunktiven. Allerdings kann es bei wiederholtem Auftreten des GBFAE zu immer ausgedehnteren Hautablösungen und zu einem schweren Krankheitsbild kommen. Als Auslöser im Kindes- und Jugendalter wurden u. a. Paracetamol, Metamizol und Sulfonamide beobachtet, aber auch chininhaltige Limonaden [21].

Das SSSS, früher auch staphylogenes Lyell-Syndrom genannt, präsentiert sich mit einer ausgedehnten Rötung ohne Flecke und Kokarden sowie ohne Schleimhauterosionen. Das Nikolski-Phänomen ist häufig positiv, bei sehr oberflächlicher Hautablösung aber „trocken“ [16]. Insgesamt ist das SSSS zwar deutlich seltener als die EN, tritt aber v. a. bei Säuglingen und Kleinkindern auf. Ursächlich ist eine Infektion mit *Staphylococcus-aureus*-Stämmen, die ein exfoliatives Toxin produzieren [14].

Auch autoimmunologisch bedingte blasenbildende Erkrankungen wie die IgA-lineare Dermatose (ggf. ausgelöst durch Vancomycin) sowie bullöse fototoxische Reaktionen müssen bei Kindern differenzialdiagnostisch in Betracht gezogen werden. Bei beiden fehlt eine Schleimhautbeteiligung. Schwere Exantheme unterschiedlicher Genese können eine trockene Desquamation nach sich ziehen, die manchmal mit der epidermalen Hautablösung bei EN verwechselt wird [16].

### Drug reaction with eosinophilia and systemic symptoms

**Figure 5:**
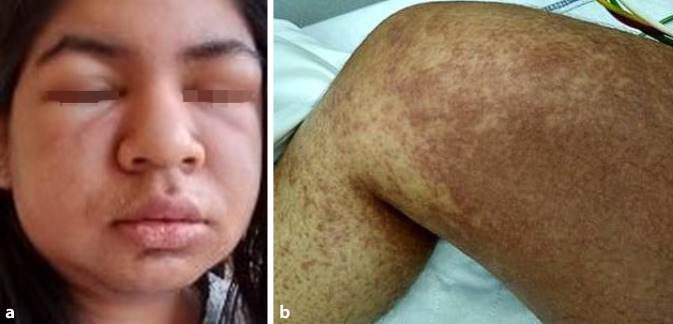

Der Hautbefund bei DRESS ist unspezifisch und sehr variabel. Nicht selten treten zu Beginn der Erkrankung makulopapulöse Hautveränderungen auf; diese lassen sich nicht von klassischen Arzneimittelexanthemen unterscheiden [9, 10]. Oftmals imponieren zuerst Rötung und Schwellung im Gesicht sowie symmetrisch am oberen Stamm oder an den oberen Extremitäten; von dort breitet sich das Exanthem über den gesamten Körper aus (Abb. 5). Diese Läsionen können sich zu infiltrierten Plaques entwickeln, zur kompletten Erythrodermie voranschreiten oder in eine exfoliative Dermatitis münden. Kokardenläsionen und purpuriforme Effloreszenzen, v. a. an den Beinen, werden ebenso beobachtet wie Urticae und eine Schleimhautbeteiligung in Form von Cheilitis, diskreten bukkalen Erosionen und gerötetem Rachen [21]. Auch sterile, follikulär und nichtfollikulär gebundene Pusteln und Spannungsblasen können vorkommen, ebenso kann ausgeprägter Juckreiz bestehen. Entscheidend für die Diagnose DRESS sind aber die Beteiligung innerer Organe sowie Veränderungen des Blutbilds. Es finden sich Lymphadenopathie, Fieber, Hepatitis, Arthralgie, interstitielle Nephritis sowie Eosinophilie und atypische Lymphozyten. Fieber von 38–40 °C, verbunden mit starkem Krankheitsgefühl, besteht bei den meisten Patienten und kann im Verlauf über einige Wochen immer wieder aufflammen [7, 9, 21].

Bei der DRESS treten nicht alle pathologischen Befunde gleichzeitig auf

Die Tatsache, dass bei DRESS nicht alle pathologischen Befunde gleichzeitig auftreten, führt nicht selten zu Schwierigkeiten in der Diagnosestellung. Zudem spiegelt die Ausprägung des Hautbefunds nicht die Schwere der Organbeteiligung wider. Als diagnostisches Hilfsmittel wurde von der RegiSCAR-Studiengruppe („International Registry for severe cutaneous adverse reactions“ (SCAR); Internationales Register für schwere kutane Unverträglichkeitsreaktionen) ein DRESS-Validierungsscore entwickelt (Tab. 2). Dieser basiert auf verschiedenen Symptomen und Laborwerten wie Fieber, Eosinophilie, Lymphadenopathie, Art und Ausmaß des Exanthems, Beteiligung innerer Organe sowie dem zeitlichen Verlauf und dem Ausschluss anderer möglicher Ursachen. Der Score umfasst die Abstufungen „kein Fall“, „möglicher“, „wahrscheinlicher“ und „sicherer“ Fall von DRESS. Für die Evaluierung der Organbeteiligung sind die Kriterien klinische Relevanz und Fehlen anderer Ursachen entscheidend. Zum Beispiel wird eine Leberbeteiligung erst ab einer Konzentrationserhöhung der Alanin-Aminotransferase um mindestens das Zweifache des Normwerts an zwei verschiedenen Messtagen ohne andere erklärbare Ursache als DRESS-Kriterium gewertet [9].

**Table 2:** 

Variable	Nein	Ja	Unbekannt
*Fieber (≥* *38,5* *°C)*	−1	0	−1
*Vergrößerte Lymphknoten (≥* *2 Körperregionen, ≥* *1* *cm)*	0	1	0
*Atypische Lymphozyten*	0	1	0
*Eosinophilie (Konzentration der eosinophilen Granulozyten)*			
700–1499/μl oder 10–19,9 %	–	1	–
≥ 1500/μl oder ≥ 20 %	–	2	–
*Hautbeteiligung*			
Ausdehnung > 50 % Körperoberfläche	0	1	0
≥ 2 der Hautveränderungen passend zu DRESS (Ödeme, Infiltration, Purpura, Schuppung)	−1	1	0
Histologische Befunde passend zu DRESS	−1	0	0
*Organbeteiligung*^*a*^			
Ein Organ	–	1	–
≥ 2 Organe	–	2	–
*Abheilung ≥* *15 Tage*	−1	0	−1
*Durchführung ≥* *3 Laboruntersuchungen*	0	1	0
Serologie/Polymerase-Kettenreaktion (Hepatitis A, B, C; EBV; CMV; Mykoplasmen/Chlamydien)			
Blutkultur			
ANA			
Mit negativem Ergebnis zum Ausschluss anderer Erkrankungen			

Endgültiger Score: < 2 kein Fall, 2 und 3 möglicher Fall, 4 und 5 wahrscheinlicher Fall, > 5 sicherer Fall

*ANA* antinukleäre Antikörper, *CMV* Zytomegalievirus, *EBV* Epstein-Barr-Virus

^a^Nach Ausschluss anderer Erkrankungen

Somit lässt sich die Diagnose DRESS – anders als bei EN – nicht allein durch Zusammenschau von klinischen Zeichen und histologischen Befunden stellen. Vielmehr sind wiederholt verschiedene klinische und laborchemische Untersuchungen erforderlich, um die Diagnose DRESS zu bestätigen. Als Basisdiagnostik sollten bei jedem Patienten mit Verdacht auf DRESS ein Differenzialblutbild, eine Kontrolle der Leber- und Nierenfunktionsparameter sowie eine Urinstatuserhebung durchgeführt werden. Darüber hinaus müssen je nach Organbeteiligung patientenspezifische Untersuchungen erfolgen und ggf. im Verlauf wiederholt werden [7, 9, 21].

Eine bilaterale Lymphadenopathie, resultierend aus einer benignen lymphoiden Hyperplasie, entwickelt sich oft im Verlauf v. a. zervikal, axillär und inguinal. Hämatologische bzw. hämatopoetische Veränderungen zeigen sich in Form einer Leukozytose mit atypischen Lymphozyten und Eosinophilie. Da die Eosinophilie mit einer Verzögerung von einer bis zwei Wochen auftreten kann, auch wenn zuvor erhöhte Leberwerte bereits wieder normal sind, muss wiederholt ein Differenzialblutbild bestimmt werden. Konzentrationen von mehr als 1,5 • 10^9^/l Eosinophilen sind für Endothelzellen toxisch und kann eine Funktionsstörung des Herzens, des Gastrointestinaltrakts, des Zentralnervensystems (ZNS), der Lungen und/oder der Nieren zur Folge haben. Auch eine transiente Hypogammaglobulinämie wurde beobachtet [7, 21].

Frühzeitig sollte aktiv nach einer kardialen Dysfunktion gesucht werden

Die Leber ist das am häufigsten betroffene innere Organ. Die Manifestationen reichen von leichter, asymptomatischer Konzentrationserhöhung der Transaminasen über Ikterus und Hepatomegalie bis zum akuten Leberversagen. Die Hepatitis kann sich, trotz Absetzens des auslösenden Arzneimittels, über Wochen hinweg verschlechtern; die Abheilung kann Monate dauern [7, 10]. An den Nieren kann sich DRESS in Form von Hämaturie, interstitieller Nephritis und akutem Nierenversagen manifestieren. Eine Myokardbeteiligung kommt eher selten vor; die Symptome sind meist unspezifisch und reichen von Tachykardie über Hypotonie, Dyspnoe zu thorakalen Schmerzen, doch wurden auch einzelne Fälle von plötzlichem Herztod beschrieben. Möglicherweise wird die Beteiligung des Herzens nicht selten übersehen, weshalb frühzeitig aktiv nach einer kardialen Dysfunktion gesucht werden sollte. Regelmäßige Blutdruck- und Frequenzkontrollen, die Bestimmung des „N-terminal prohormone of brain natriuretic peptide“ (NT-proBNP) und ggf. die Durchführung einer Echokardiographie- oder einer Magnetresonanztomographie(MRT)-Untersuchung des Herzens sind auch beim Fehlen einer Eosinophilie angezeigt [21].

Weitere Organmanifestationen können die Lungen (akutes Atemnotsyndrom, Dyspnoe, interstitielle Pneumonitis, pathologische Lungenfunktion, trockener Husten, Vaskulitis), das ZNS (aseptische Meningitis, Enzephalitis, Koma, Krampfanfälle, Sprachstörungen) sowie Gelenke (Arthralgie) und Muskeln (Myositis) betreffen. In Einzelfällen wurden Ösophagitis, Pankreatitis und Kolitis beschrieben; diese gehen mit abdominellen Schmerzen oder blutigen Diarrhöen einher, weshalb bei Verdacht auf eine Beteiligung des Verdauungstrakts eine endoskopische Untersuchung sinnvoll ist [21].

Da die Hautveränderungen bei DRESS so variabel sind, ist auch das histologische Bild vielfältig. Meist finden sich dichte lymphozytäre Infiltrate mit eingestreuten Eosinophilen in der oberflächlichen Dermis und/oder perivaskulär. Auch können dermale Ödeme bestehen, sowie ein bandförmiges Infiltrat aus atypischen Lymphozyten mit Epidermotropismus, das an eine Mycosis fungoides erinnert. Die Hautbiopsie bei V. a. DRESS dient v. a. zum Ausschluss anderer Erkrankungen [32].

Japanische Kollegen haben einen engen Zusammenhang zwischen dem Wiederaufflammen von Fieber, Hepatitis und dem Nachweis einer Reaktivierung des humanen Herpesvirus 6 (HHV 6) beobachtet [21]. Daher empfehlen sich entsprechende serologische Untersuchungen (Nachweis von spezifischen IgM, deutlich erhöhtem IgG oder HHV6-DNA in Leukozyten), denn bei nachgewiesener Virusreaktivierung muss mit einem progredienten Verlauf, wiederholtem Aufflammen und/oder verzögerter Abheilung gerechnet werden.

Die häufigste Differenzialdiagnose von DRESS bei Kindern und Jugendlichen sind makulopapulöse Exantheme unterschiedlicher Genese, z. B. infektions- oder arzneimittelinduziert oder eine Kombination aus beidem (allgemein bekannt für Aminopenicilline und Epstein-Barr-Virus [EBV]). Neben den Hautausschlägen kann es auch zur Konzentrationserhöhung der Transaminasen kommen, wobei aber andere Organmanifestationen von DRESS fehlen. Eine wichtige Differenzialdiagnose stellt das Kawasaki-Syndrom oder im Zusammenhang mit einer zurückliegenden Infektion mit dem „severe acute respiratory syndrome coronavirus 2“ (SARS-CoV-2) auch das Pediatric Inflammatory Multisystem Syndrom (PIMS) dar; diese können ebenfalls mit hohem Fieber, Exanthem, Schleimhautbeteiligung (Bindehaut, Lippen, Zunge) und Lymphknotenschwellungen einhergehen [7, 21]. Aufgrund von Ödemen der Haut treten bei DRESS nicht selten Blasen, die an eine EN denken lassen, auf [21].

## Auslösende Faktoren

### Epidermale Nekrolyse

Zur Bewertung des Risikos von Arzneimitteln, eine EN auszulösen, wurden in Europa 2 multizentrische Fall-Kontroll-Studien durchgeführt. Diese schlossen Patienten aller Altersgruppen, die eine EN im häuslichen Umfeld (außerhalb des Krankenhauses) entwickelten und deren Reaktion zur stationären Aufnahme führte, ein [17]. Die Daten der in diese beiden Studien eingeschlossenen Kinder wurden in einer Metaanalyse separat untersucht. Hier ließ sich ein deutlich erhöhtes Risiko für bestimmte Antiepileptika (Carbamazepin, Lamotrigin, Phenobarbital), antibakterielle Sulfonamide, einschließlich Sulfasalazin, nachweisen. Demgegenüber war das Risiko für verschiedene Antibiotikagruppen deutlich niedriger (Cephalosporine, Makrolide, Aminopenicilline [11]). Eine Untersuchung von Kindern mit EN, die in einem amerikanischen Verbrennungszentrum versorgt wurden, kam zu ähnlichen Ergebnissen [22]. Allopurinol als häufigster Auslöser von EN (und von DRESS) bei Erwachsenen spielt bei Kindern kaum eine Rolle [11, 17].

Bevor allerdings im Einzelfall das mögliche auslösende Agens identifiziert werden kann, gilt es, den Tag des Beginns der Reaktion, den sog. Indextag, festzulegen. Hierfür muss der genaue zeitliche Verlauf hinsichtlich des Auftretens der einzelnen klinischen Symptome bekannt sein, denn der Beginn der Reaktion ist meist nicht erst der Tag der Blasenbildung, sondern bereits zuvor bestehende Zeichen wie Rötung der Haut oder unspezifische Prodromalsymptome markieren den Reaktionsbeginn.

Zwischen dem Einnahmebeginn des auslösenden Arzneimittels und dem Indextag liegen meist ein bis vier Wochen

**Figure 6:**
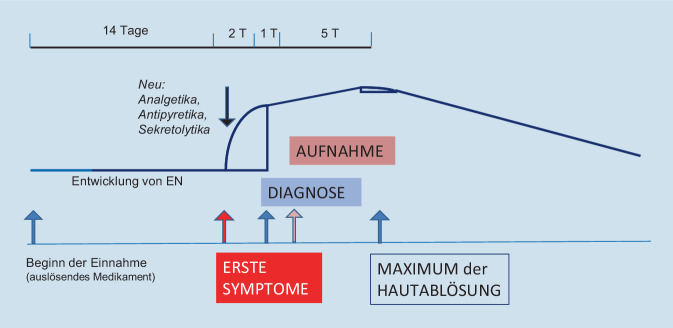

Zu diesen Prodromalsymptomen gehören Unwohlsein, Kopfschmerzen, Halsweh, aber auch Fieber und Schüttelfrost. Sie treten oft einen bis drei Tage vor den ersten sichtbaren Zeichen an Haut und Schleimhaut bzw. mehrere Tage vor der Blasenbildung auf (Abb. 6). Nicht selten werden zur Behandlung dieser Symptome fiebersenkende, schmerzstillende und schleimlösende Substanzen verabreicht, die dann als Auslöser angeschuldigt werden, wenn Blasen der Haut und/oder Erosionen der Schleimhaut hinzukommen (*protopathischer Bias*). Allerdings sind die zur Behandlung der ersten Symptome der schweren Hautreaktion gegebenen Arzneimittel nicht als ursächlich anzusehen, v. a. wenn sie bereits früher angewendet und vertragen wurden [20, 23]. Vielmehr werden die Auslöser von EN im Rahmen des ersten Einnahmezyklus angewendet und die durchschnittliche Zeitspanne zwischen Einnahmebeginn des auslösenden Arzneimittels und Reaktionsbeginn beträgt eine bis vier Wochen [17, 21].

Im dZh wurden keine Rezidive von infektionsinduzierten EN-Fällen beobachtet

Bereits in den oben genannten Studien zeigte sich, dass jeweils etwa 65 % der EN-Patienten im relevanten Zeitraum vor der Reaktion ein Arzneimittel mit signifikantem Risiko eingenommen hatten [17]. Dies konnte auch in der RegiSCAR-Studie bestätigt werden; diese beruht auf einem multinationalen Fallregister, in dem jeder einzelne validierte EN-Fall mithilfe eines spezifischen Algorithmus hinsichtlich der auslösenden Faktoren analysiert wird [27]. In knapp 30 % der Fälle ließ sich kein medikamentöser Auslöser bestimmen. Die Daten des dZh (entsprechend dem deutschen Anteil der RegiSCAR-Studie) ergeben, dass bei Kindern mit EN in nicht mehr als 50 % der Fälle ein medikamentöser Auslöser identifiziert werden kann. In vielen Fällen besteht als möglicher Trigger-Faktor ein Infektionsgeschehen im relevanten Zeitraum vor Reaktionsbeginn. Oftmals wird klinisch eine Infektion diagnostiziert, doch lässt sich trotz ausgedehnter Labordiagnostik kein bestimmter Erreger nachweisen. Im Gegensatz zum EEMM sind es aber weder Herpes-simplex-Eruptionen noch Mykoplasmeninfektionen, die EN induzieren, sondern eher andere virale Erreger, mit Ausnahme von SARS-CoV‑2. Im dZh wurden keine Rezidive von infektionsinduzierten EN-Fällen beobachtet, möglicherweise weil die auslösenden Viren sich rasch verändern und nicht wiedererkannt werden. Andere Fälle müssen als idiopathisch betrachtet werden [16].

### Drug reaction with eosinophilia and systemic symptoms

Auch bei DRESS stützt sich die Risikobewertung von Arzneimitteln in erster Linie auf klinisch-epidemiologische Daten. In einer großen Fallserie von strikt validierten DRESS-Fällen wurden in knapp 40 % der Fälle Allopurinol und Carbamazepin als auslösende Faktoren identifiziert [10]. Bei Kindern und Jugendlichen wurden v. a. Antiepileptika wie Carbamazepin (und auch Oxcarbazepin), Lamotrigin oder Phenobarbital als Auslöser beobachtet, aber auch Sulfasalazin, Dapson, Vancomycin und Minocyclin [7, 10]. Typisch für DRESS ist die lange Latenzzeit vom Einnahmebeginn des Arzneimittels bis zum Auftreten der Erkrankung von mehreren Wochen (meist 2 bis 8, manchmal sogar 12 Wochen). DRESS tritt ebenfalls vorzugsweise im ersten Einnahmezyklus einer Substanz auf, anscheinend unabhängig von der eingenommenen Dosis [10]. Das Akronym DRESS impliziert bereits Arzneimittel als auslösenden Faktor („drug reaction“), wobei unklar ist, ob es möglicherweise doch Fälle gibt, die nicht medikamenteninduziert, sondern evtl. infektionsgetriggert sind.

## Pathogenetische Faktoren

### Epidermale Nekrolyse

Die akute Keratinozytennekrose bei EN wird auf einen ausgedehnten apoptotischen Prozess zurückgeführt. Zytotoxische T‑Zellen können die Apoptose initiieren, verstärkt durch das Freisetzen von Perforinen und Zytokinen, wie z. B. Tumor-Nekrose-Faktor(TNF)-α oder Granzym B, Proteine wie Fas-Antigen (CD95) und p55-TNF-α-Rezeptor. Allerdings sind nicht Fas und Fas-Ligand die wichtigsten Zytokine in der Akutphase von EN, sondern das kationische Protein Granulysin [6]. In genetischen Untersuchungen konnte gezeigt werden, dass eine Prädisposition für EN vorliegt, die sowohl für bestimmte Arzneimittel als auch für ethnische Faktoren spezifisch ist. Zum Beispiel ließ sich der bei Han-Chinesen nachgewiesene hochsignifikante Zusammenhang von carbamazepininduzierter EN und HLA-B*1502 bei europäischen Patienten nicht bestätigen [12]. Offenbar ist EN eine sehr arzneimittelspezifische Reaktionsform, d. h., ein bestimmter genetisch prädisponierter Patient reagiert nur auf ein spezifisches Arzneimittel mit einer EN; dieses sollte künftig gemieden werden. Untersuchungen zum genetischen Muster von infektionsinduzierten EN-Fällen wurden bislang nicht systematisch durchgeführt. Allerdings scheinen einige Berichte über spezifische HLA-Allele in Fällen, die vermeintlich durch Antipyretika und Sekretolytika ausgelöst wurden, letztlich im Zusammenhang mit infektionsinduzierten Reaktionen zu stehen [23].

### Drug reaction with eosinophilia and systemic symptoms

Verschiedene immunologische Mechanismen sind an der Entstehung von DRESS beteiligt, z. B. die Aktivierung von CD4^+^- und CD8^+^-T-Zellen, die zur Freisetzung verschiedener Zytokine mit zytotoxischem Potenzial führen und Entzündungen hervorrufen können. Weiterhin scheint die Ausschüttung von Interleukin 5 wichtig; Interleukin 5 fördert die vermehrte Bildung von Eosinophilen, einem wesentlichen Charakteristikum von DRESS [7, 9, 21].

Inwiefern die bereits erwähnte Virusreaktivierung eine pathogenetische Rolle spielt, ist noch nicht abschließend geklärt. Einerseits könnte die starke Immunstimulation im Rahmen der Erkrankung ursächlich für die Reaktivierung der lymphotropen Viren sein. Andererseits könnte aber auch die Virusreaktivierung selbst einen zusätzlichen Stimulus für das Immunsystem, der mit einem protrahierten Krankheitsverlauf einhergeht, darstellen.

Es wurden auch verschiedene genetische Veranlagungen beobachtet und diskutiert. Bei einigen Patienten konnte ein Defekt der Epoxidhydroxylase nachgewiesen werden; eines Enzyms, das an der Metabolisierung von Arzneimitteln, v. a. von Antiepileptika, beteiligt ist und dessen Veränderung zur Akkumulation toxischer Metaboliten führt [21].

## Therapeutische Maßnahmen

### Epidermale Nekrolyse

Bei ausgedehnter Hautablösung von etwa 30 % der Körperoberfläche (entsprechend etwa einer Epidermisablösung des gesamten ventralen und dorsalen Stamms), ist eine intensivmedizinische Versorgung der betroffenen Patienten notwendig. Das gilt auch für Kinder und Jugendliche. Hierfür kommen pädiatrische Intensivstationen, idealerweise Verbrennungseinheiten, in Betracht. Die supportive Therapie ist unerlässlich und schließt die Betreuung durch ein multidisziplinäres Team ein [13, 16]. Zu den symptomatischen Maßnahmen gehören die Erhöhung der Raumtemperatur auf 30–32 °C, die Lagerung auf Metalline-Folie (Lohmann & Rauscher GmbH & Co. KG, Neuwied, Deutschland) bzw. in einem Luftkissenbett und die i.v.-Flüssigkeitszufuhr mit isotonischer Elektrolytlösung, ggf. Albuminsubstitution, entsprechend intensivmedizinischer Behandlungsprotokolle. Dabei gilt es zu berücksichtigen, dass EN-Patienten einen im Vergleich zu Verbrennungspatienten verminderten Flüssigkeitsbedarf haben (ca. zwei Drittel bis drei Viertel des Bedarfs von Verbrennungsopfern). Um den Proteinverlust zu minimieren, aber auch um eine intestinale Atonie zu vermeiden, wird eine frühzeitige Ernährung über eine Nasensonde empfohlen [8, 16].

Auf eine breite antibiotische Abdeckung ohne Verdacht auf eine Infektion, d. h. auf eine rein prophylaktische Antibiotikagabe, sollte verzichtet werden, v. a. weil Infektionszeichen verschleiert werden können. Dagegen sollten Antibiotika gezielt nach Antibiogramm nur dann eingesetzt werden, wenn Zeichen einer Infektion oder Septikämie vorliegen. Auch eine adäquate Schmerz- und ggf. sedierende Therapie sind notwendig [8].

Zur Lokalbehandlung werden antiseptische Lösungen oder Gele empfohlen (z. B. Octenidin, Chlorhexidin, Polihexanid); auf belastete Körperareale kann wirkstofffreie, nichtklebende Netzgaze aufgebracht werden. Zum Teil noch pralle Blasen sollten aseptisch eröffnet werden, wobei die nekrotische Epidermis belassen wird, um ein zu starkes Austrocknen der Haut zu verhindern und so die Reepithelisierung zu fördern [8, 13].

Alle Patienten mit einer EN sollten rasch augenärztlich untersucht werden

Das in früheren Jahren von manchen Verbrennungsmedizinern propagierte aggressive Bürstendébridement ist weitgehend verlassen worden, da das Risiko der Narbenbildung bei einer ansonsten nicht zur Vernarbung führenden subepidermalen Blasenbildung deutlich erhöht ist. Ein vorsichtiges Entfernen nekrotischer Epidermis durch ein desinfizierendes, sog. Verbrennungsbad und anschließendes Aufbringen von Wundverbänden hingegen wird besser toleriert [15, 16].

Für die Behandlung der erosiven Mundschleimhautbeteiligung eignen sich milde antiseptische Mundspülungen; auf betroffene Lippen kann dexpanthenolhaltige Salbe appliziert werden. Auch für die Behandlung von genitalen oder analen Erosionen kommen antiseptische Lösungen, ebenfalls in Form von Sitzbädern, und Cremes zur Anwendung, die u. a. das Verkleben der erosiven Schleimhäute verhindern sollen. Alle Patienten mit einer EN sollten rasch augenärztlich untersucht werden. Wird eine Augenbeteiligung mit Konjunktivitis, Blepharitis und/oder Kornea-Erosionen diagnostiziert, ist die tägliche Mitbetreuung durch einen erfahrenen Augenarzt notwendig. Neben antiseptischen oder antibiotischen bzw. steroidhaltigen Augentropfen muss bei vielen Patienten eine Symblepharon-Prophylaxe durch konsequente Lidrandpflege oder das Einlegen von Illig-Schalen in Betracht gezogen werden, um mögliche schwere Folgeschäden zu vermeiden [5]. Spätere chirurgische Maßnahmen, wie das Lösen von Synechien oder Schleimhauttransplantationen, wie z. B. Konjunktivenersatz durch Mundschleimhautanteile oder Transplantation von Limbuszellen, können die Problematik meist nicht langfristig lösen. Bei Patienten, die ein Sicca-ähnliches Syndrom entwickeln, kann künstliche Tränenflüssigkeit zu einer Verbesserung der Situation führen [5, 8, 16].

Über geeignete immunmodulierende Substanzen wird nach wie vor kontrovers diskutiert. In zwei unabhängigen systematischen Reviews mit Metaanalysen konnte weder auf Studien- noch auf individueller Patientenebene ein positiver Effekt von intravenösen Immunglobulinen (IVIG) hinsichtlich der Letalität nachgewiesen werden [21]. In einer der Metaanalysen wurde für die kurzzeitige Gabe von systemischen Glukokortikosteroiden im Hinblick auf das Überleben ein positiver Effekt festgestellt [33]. Die vorliegenden großen Beobachtungsstudien schließen Kinder und Erwachsene ein, doch ist der prozentuale Anteil der Kinder aufgrund der epidemiologischen Verteilung der Krankheit in verschiedenen Altersgruppen gering, sodass Subgruppenanalysen für die pädiatrischen Altersgruppen fehlen.

Basierend auf der aktuellen Studienlage gibt es für den Einsatz von Cyclosporin A die beste Evidenz, und zwar sowohl hinsichtlich einer geringeren Letalität (gemessen am Score of Toxic Epidermal Necrolysis [SCORTEN]) als auch in Bezug auf eine frühere Reepithelisierung [19, 24]. Beim SCORTEN handelt es sich um ein Instrument zur Prognoseeinschätzung von EN, das innerhalb der ersten Tage im Verlauf der Reaktion angewendet werden sollte. Jeder der sieben unabhängigen Faktoren erhält das gleiche Gewicht, wobei die Überlebenschance für den betroffenen Patienten mit Zunahme der Score-Werte fällt (Tab. 3 [2, 4]). Während zunächst nur Daten zu Patienten im Alter von 16 bis 66 Jahren vorlagen, gab es bald auch Berichte über die Therapie mit Cyclosporin A bei Kindern mit einer EN [30]. Mittlerweile bestehen im dZh ausgedehnte Erfahrungen mit Cyclosporin A zur immunmodulierenden Therapie von vielen Kindern mit EN, die sehr positiv sind.

**Table 3:** 

Variable	Vorliegend	Score-Wert
Patientenalter (≥ 40 Jahre)	Ja	1
Herzfrequenz (≥ 120/min)	Ja	1
Maligne Grunderkrankung	Ja	1
Ablösung der Körperoberfläche am 1. Tag	≥ 10 %	1
Serum-Harnstoff-Konzentration (≥ 10 mmol/l)	Ja	1
Serum-Bikarbonat-Konzentration (< 20 mmol/l)	Ja	1
Serum-Glucose-Konzentration (≥ 14 mmol/l)	Ja	1

Mögliches Scores 0–7: Mit steigendem Punktwert verschlechtert sich die Prognose des Patienten

Zusammenfassend lässt sich zur immunmodulierenden Therapie von EN Folgendes konstatieren:

1. Intravenöse Immunglobuline werden nicht für die Therapie von EN empfohlen.
2. Glukokortikosteroide scheinen sich, kurzzeitig (1–2 mg/kgKG für 3 Tage) verabreicht, positiv auszuwirken (v. a. auf die schmerzhafte geschwollene Schleimhaut und das Gesamtbefinden).
3. Die besten Ergebnisse liegen für Cyclosporin A vor, das in einer Dosierung von 3(–5) mg/kgKG und Tag für insgesamt 10 Tage verabreicht werden sollte, sofern in den letzten 24 h eine Progression der Hautreaktion (neue Erytheme und neue Blasen) festzustellen war. Die Dosis sollte in 2 Tagesdosen gegeben und ggf. an eine eingeschränkte Nierenfunktion adaptiert werden [24].

### Drug reaction with eosinophilia and systemic symptoms

Die wichtigste Maßnahme bei DRESS besteht in der frühzeitigen Identifizierung und dem Absetzen des auslösenden Arzneimittels. Zu berücksichtigen ist die lange Latenzzeit zwischen Einnahmebeginn und dem klinischen Auftreten von DRESS, die oft mehrere Wochen beträgt [10].

In Ermangelung evidenzbasierter Studiendaten stellt die supportive Therapie aktuell den Goldstandard dar. Zur topischen Therapie der Haut wird die Applikation von hochpotenten steroidhaltigen Externa empfohlen [7]. Bei starkem Juckreiz können systemische Antihistaminika lindernd wirken. Sollte sich eine exfoliative Dermatitis entwickeln, ist eine Therapieerweiterung um Erhöhung der Umgebungstemperatur, Ausgleich der Elektrolytverschiebung und Sepsisprävention notwendig [7, 21].

Der Einsatz systemischer Glukokortikosteroide wird oftmals bei Beteiligung innerer Organe befürwortet. Die empfohlene Dosierung beträgt zwischen 0,5 und 2,0 mg Prednisolonäquivalent/kgKG [21]. Nicht in allen Fällen ist diese Therapieoption effektiv, während sie in anderen Fällen zu einer schnellen Verbesserung von klinischen Symptomen und Laborwerten führt. Die Therapiedauer richtet sich nach der klinischen Symptomatik und kann von einer Woche bis zu fünf Monaten betragen. Ein Wiederaufflammen der Reaktion während der Steroidreduktion wurde beobachtet, weshalb ein langsames Ausschleichen zu empfehlen ist [7, 21].

## Prognose, Komplikationen und Folgeschäden

### Epidermale Nekrolyse

Die Prognose von erwachsenen und insbesondere älteren Patienten mit ausgedehnter bullöser Arzneimittelreaktion ist insgesamt ungünstig, und das Sterberisiko ist hoch. Bei Kindern und Jugendlichen ist die Prognose bedeutend besser [22]. Mithilfe des SCORTEN lässt sich im Einzelfall – für Kinder und für Erwachsene – eine prognostische Aussage treffen (Tab. 3 [2, 4]). Im Verlauf von EN kann es zur Begleithepatitis, tubulären Nephritis oder tracheobronchialen Schleimhautbeteiligung kommen; diese bilden sich in der Mehrzahl der Fälle aber relativ rasch zurück. Häufige Komplikationen sind nosokomiale Infektionen und Sepsis, die nicht selten über zentrale Venenzugänge hervorgerufen werden. Daher sollten nach Möglichkeit periphere Zugänge gewählt und spezifische Hygienemaßnahmen wie Umkehrisolation etc. eingehalten werden [8, 22].

Langfristige Folgeerscheinungen unterschiedlichsten Schweregrads treten bei der Mehrzahl der überlebenden Patienten auf und betreffen v. a. Haut und Schleimhäute [16, 31]. Während die Hautläsionen bei EN narbenlos abheilen, bestehen als Folge der Entzündungsreaktion oft über Monate bis Jahre Hyper- und Hypopigmentierungen der Haut. Auch ein reversibles Effluvium sowie ein vollständiger Nagelverlust werden beobachtet. Problematischer sind Verwachsungen im Bereich der Schleimhäute, die zu Strikturen z. B. der Urethra oder des Ösophagus führen können [31]. Die sicherlich gefährlichste und für die Patienten dramatischste Folgeerscheinung ist die Symblepharon-Bildung mit Entropium und Trichiasis, die zur Erblindung führen kann [5]. Hierdurch kann es bei Kindern u. a. zu Entwicklungsstörungen kommen. Auch Albträume kommen vor. Allerdings ist die Einschätzung der Häufigkeit solcher Beschwerden schwierig, da die meisten Verlaufsuntersuchungen sich auf Patienten ab 18 Jahren beziehen.

### Drug reaction with eosinophilia and systemic symptoms

Bei DRESS wurden an verschiedenen Organen Folgeschäden beschrieben. Hierzu gehören z. B. eine hypothyreote Stoffwechsellage einen bis zwei Monate nach der Erkrankung bei zuvor euthyreoten Patienten, die sich im Verlauf einiger Monate zurückbildete. Auch Hyperthyreose und M. Basedow wurden beobachtet; diese äußerten sich klinisch in Form von Palpitationen, Erregbarkeit und Schlafstörungen, wobei längere Beobachtungen ausstehen. Spezifische Langzeituntersuchungen bei Kindern mit DRESS liegen nicht vor [7, 10].

Wiederholt konnte im zeitlichen Abstand von drei Wochen bis 10 Monaten nach Erkrankungsbeginn das Auftreten eines Diabetes mellitus beobachtet werden [21]. Ob es sich um eine transiente Stoffwechselstörung oder eine lebenslange Folgeerscheinung handelt, ist aktuell aufgrund fehlender Langzeituntersuchungen ungewiss. Das therapeutische Konzept sollte zur Vermeidung bzw. frühzeitigen Behandlung von möglichen Folgeerscheinungen regelmäßige Verlaufskontrollen einschließen. Kontrollen von Blutzucker- und Schilddrüsenwerten sowie Leber- und Nierenfunktionsparametern sind ebenso zu empfehlen wie Blutbild‑, Leber- und Nierenwertkontrollen [7, 21].

## Fazit für die Praxis

- Schwere kutane Arzneimittelreaktionen wie EN und DRESS treten auch bei Kindern und Jugendlichen, wenngleich seltener als bei Erwachsenen auf.
- Da sich die Hauteffloreszenzen im Verlauf der Erkrankungen wandeln und sich Blasen und Erosionen ausbreiten können, ist eine gute Fotodokumentation zu Beginn und im Verlauf der Erkrankung ratsam.
- Auslösende Arzneimittel bei Kindern und Jugendlichen sind v. a. Antiepileptika und antibakterielle Sulfonamide sowie Sulfasalazin, wobei die Expositionszeit bei DRESS länger ist als bei EN. Bei EN ist nur in etwa der Hälfte der Fälle ein Arzneimittel der auslösende Faktor; ein großer Teil der Reaktionen erscheint infektionsinduziert.
- Um den Auslöser der Reaktion festzustellen und nicht Medikamente anzuschuldigen, die zur Behandlung von Prodromalsymptomen gegeben wurden, muss der Beginn der Reaktion korrekt bestimmt werden. Lässt sich ein medikamentöser Auslöser identifizieren, sollte ein entsprechender Allergiepass ausgestellt werden.
- Die supportive Therapie stellt den Goldstandard der Behandlung dar. Als immunmodulierende Therapie bei EN können kurzfristig Glukokortikosteroide eingesetzt werden; Cyclosporin A ist bei Progression der Reaktion zu bevorzugen. Bei DRESS sollten Glukokortikosteroide über längere Zeit gegeben und langsam ausgeschlichen werden.
- Ein interdisziplinäres Team ist in der Akutphase der Erkrankung und im Verlauf notwendig, um Folgeschäden zu vermeiden bzw. frühzeitig zu erkennen und adäquat zu behandeln. Folgeschäden manifestieren sich bei EN v. a. an den Schleimhäuten und betreffen bei DRESS eher das endokrine System.
